# Muscle-Nerve-Nerve Grafting Improves Facial Reanimation in Rats Following Facial Nerve Injury

**DOI:** 10.3389/fneur.2021.723024

**Published:** 2021-12-08

**Authors:** Steven J. Charous, Michael J. Hutz, Samantha E. Bialek, Jane K. Schumacher, Eileen M. Foecking

**Affiliations:** ^1^Department of Otolaryngology–Head and Neck Surgery, Loyola University of Chicago, Maywood, IL, United States; ^2^Research Service, Edward Hines Jr. VA Hospital, Hines, IL, United States; ^3^Stitch School of Medicine, Loyola University, Maywood, IL, United States

**Keywords:** interpositional grafting, muscle-nerve-muscle neurotization, facial nerve injury, muscle-nerve-nerve neurotization, functional recovery

## Abstract

Nerve injury resulting in muscle paralysis from trauma or surgery is a major medical problem. Repair of such injuries with existing nerve grafting and reconstructive techniques often results in less than optimal outcomes. After previously demonstrating significant return of function using muscle-nerve-muscle (MNM) grafting in a rat facial nerve model, this study compares a variant of the technique, muscle-nerve-nerve (MNN) neurotization to MNM and interposition (IP) nerve grafting. Thirty male rats were randomized into four groups (1) control with no intervention, (2) repair with IP grafts, (3) MNM grafts and (4) MNN grafts. All groups had the buccal and marginal mandibular branches of the right facial nerve resected. Return of vibrissae movement, orientation, and snout symmetry was measured over 16 weeks. Functional recovery and muscle atrophy were assessed and quantified. All interventions resulted in significant improvement in vibrissae movement and orientation as compared to the control group (*p* < 0.05). The MNM and MNN groups had significantly less time to forward vibrissae movement as compared to controls (*p* < 0.05), and a large number of animals in the MNN group had coordinated vibrissae movement at 16 weeks. MNN and IP grafts retained significantly more muscle mass as compared to control (*p* < 0.05). Thus, MNN grafting is a promising adjuvant or alternative technique for reanimation for patients with unilateral peripheral nerve injury who are not candidates for primary neurorrhaphy.

## Introduction

Nerve injuries resulting in muscle paralysis are usually a result of trauma or surgery and represent a major medical problem. Existing nerve grafting and reconstructive techniques for the repair of such injuries can result in less than ideal functional outcomes with synkinesis and unpaired movement. Immediate coaptation of the severed nerve is the optimal solution, but when this is not feasible, other strategies are necessary to induce restoration of muscle function. Such techniques include, nerve grafts, splitting nerves longitudinally to share fascicles with the denervated muscle, end-to-side grafting, nerve-muscle pedicles, and direct muscular neurotization by implanting the distal end of a nerve into denervated muscle (1–5).

Muscle-nerve-muscle (MNM) grafting uses an autogenous nerve graft that serves as a conduit pairing an innervated, normally functioning muscle with a denervated muscle. After interposing the harvested graft between the muscles, axonal sprouting is induced in the normal muscle and traverses the graft to innervate the denervated muscle. Thus, when the normal muscle is stimulated, simultaneous contraction of the paired, denervated muscle is observed. MNM grafting has the advantages of being relatively simple technically and having minimal associated risk or morbidity. This technique has been described to be effective in rat facial nerve and somatic nerve models, a dog laryngeal nerve model, and in a limited number of human facial nerve patients (5–8). We previously demonstrated the feasibility and comparable results of this grafting technique to other nerve grafting techniques, and the potential of using multiple grafts in order to try to “amplify” the nerve signal and improve results further (5, 9).

This paper is the first to describe a variant of the MNM model in which one end of the nerve conduit is embedded into the normal muscle and the other end is anastomosed to the severed distal nerve that supplied the denervated muscle. We hypothesized that this new MNN group would have improved functional movement, decreased muscle atrophy, and histologic evidence of increased reinnervation compared to controls and MNM grafted groups. Further, the MNN group would have improved innervation by utilizing the original intact nerve-muscle junctions of the denervated muscle and would be more effective than the MNM technique of embedding the nerve into the affected muscle and awaiting new nerve-muscle junctions to form. We also compared to the MNN to the “gold” standard of direct nerve coaptation. The potential applications of such a technique in treating facial nerve paralysis, paralysis of the larynx, and other unexplored areas are great.

## Materials and Methods

### Animals

Thirty male Sprague-Dawley rats (200 g) from Envigo (Indianapolis, Indiana, USA) were housed under a 12-h light/dark cycle and received a standard rodent diet and water ad libitum. All surgical procedures were completed in accordance with the National Institutes of Health guidelines on care and use of laboratory animals for research purposes and approved by the institutional animal care and use committee at Edward Hines Jr. VA Hospital.

Animals were randomly assigned to one of four groups: no graft (CTL) (negative control), interposition (IP) graft, MNM graft, and MNN graft. All animals then underwent a right transfacial approach with concurrent parotidectomy. The buccal and marginal mandibular branches were immediately identified deep to the subcutaneous tissues. Retrograde dissection of these branches allowed for identification of the main trunk of the facial nerve. The buccal and marginal mandibular facial nerve branches were harvested from their initial ramification to their distal insertion into the muscles of the vibrissae, yielding ~2.0 cm segments, and were subsequently used as the nerve grafts. The incision was extended across the snout to expose the contralateral vibrissae muscle pad in all groups.

**Figure 1:**
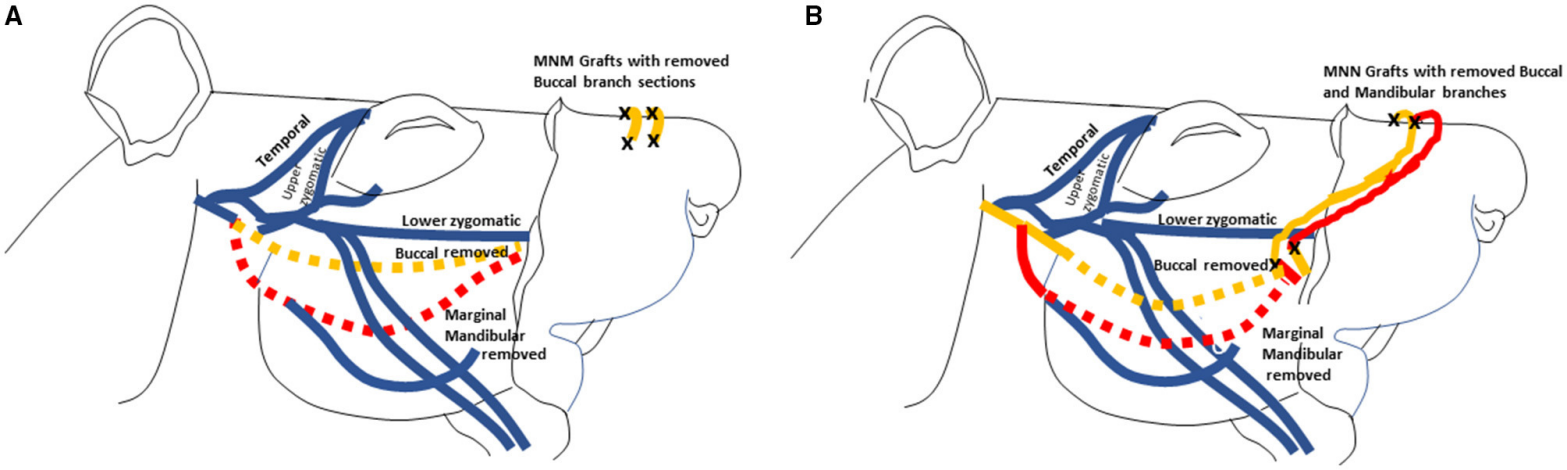
Illustrations depicting of the MNM grafting technique with two grafts **(A)**, and the MNN **(B)** grafting technique are displayed. These images were adapted from drawings with permission from Braintree Scientific, Inc. (10). The buccal branch is displayed in orange and the marginal mandibular branch is in red. The dashed nerve lines represent when the nerves was removed. The black X demonstrates all the places where the nerve grafts were sutured.

The procedures are schematically depicted in Figure 1. Dashed lines represent nerves that were removed and the black “X” demonstrates where the nerve graft was sutured (adapted with permission from Braintree Scientific, Inc.) (10). The buccal (orange) and mandibular (red) branches were removed from the control animals with no further intervention as previously depicted (9).

### Interposition (IP) Graft Repair

The buccal and marginal mandibular facial nerve branches were dissected and harvested as previously described (5). The buccal nerve graft served as the graft for the mandibular branch and was sutured to the nerve stumps of the mandibular branch. The mandibular nerve graft served as the graft for the buccal branch and was sutured to the nerve stumps of the buccal branch with 9-0 nylon sutures through the epineurium as previously depicted (9).

### Muscle-Nerve-Muscle Grafting Repair

In the group that had the MNM repair technique, the buccal and mandibular nerve branches were harvested and sewn from the denervated right levator labii superioris muscle pad into the innervated left levator labii superioris muscle pad as previously described (5). The two nerve grafts were tunneled ~1–2 mm into superior portion of the right levator labii superioris muscle parallel to the muscle fibers, and secured with 9-0 nylon sutures through the epineurium (Figure 1A). An incision was then made in the innervated left levator labii superioris muscle bed where the graft was embedded to introduce trauma to the axons in the muscle pad to induce axonal sprouting into and across the grafts.

### Muscle-Nerve-Nerve Grafting Repair

In the group undergoing MNN repair, the buccal, and mandibular nerve branches were harvested and the distal ends of the two nerve grafts were implanted into the superior portion of the left unaffected levator labii superioris muscle, parallel to the muscle fibers, The proximal end of the buccal branch were sutured to the nerve stump of the mandibular branch, and the proximal end of the mandibular branch was sutured to the nerve stump of the buccal branch (Figure 1B).

### Functional Assessments

Animals were observed weekly for 16 weeks to assess functional recovery of vibrissae movement and orientation and snout symmetry. This time frame was chosen based on our previous study that demonstrated a large proportion of animals experienced some intervention dependent functional recovery by 16 weeks (5, 9). Recovery of facial nerve function on the right denervated side was compared to the innervated left side. All functions were assessed by two laboratory technicians in a blinded manner. Vibrissae movement was assessed utilizing a 6-point scale as previously described (9). Briefly, 1 represented no movement, 2 represents vibration, 3 represented the onset of whisking movement, 4 represented forward but delayed whisking, 5 represented forward coordinated movement with the innervated side of unequal intensity, and 6 represented a forward coordinated movement with equal intensity to the innervated side. Vibrissae orientation was assessed on a 3-point scale, where 1 represented vibrissae on the denervated side flattened against the face, 2 represented vibrissae that are less flattened and oriented more forward, but not matching the innervated side, and 3 represented vibrissae on the denervated side indistinguishable from the innervated side. Symmetry of the snout from the midline was quantified on a 4-point scale, where 1 represented minimal symmetry (~45-degree deflection from midline), 2 represented mild symmetry (30 degrees from midline), 3 represented moderate symmetry (15 degrees from midline), and 4 represented complete symmetry.

### Muscle Weights

At the end of the 16-week experiment, animals were euthanized by isoflurane overdose. The denervated and innervated mystacial vibrissae muscle pads, containing the levator labii superioris, dilator naris, nasolabialis profundus, and the maxiolabialis, were dissected out from the nasal bone through the premaxillary bone and weighed [anatomy described by Haidarliu et al. (11)]. Muscle atrophy was calculated as a standardized percentage of the denervated vibrissae muscle pad weight to the innervated vibrissae muscle pad weight.

### Statistical Analysis

Significant changes in vibrissae movement, orientation, and nose symmetry were determined using a two-way analysis of variance [ANOVA; factors = time (days post-operative) and treatment], followed by a Newman Keuls' multiple comparison *post-hoc* test. Significant changes in muscle weights amongst the groups were determined using one-way ANOVA followed by Tukey's multiple comparison test (GraphPad Prism). All data is represented as Mean ± SEM An a priori repeated measures ANOVA (within-between interaction) power analysis was run (effect size f = 0.24, α = 0.05, power = 0.95) using G^*^Power 3.1 determined the total sample size for the 4 groups was 28.

## Results

### Functional Recovery

**Figure 2:**
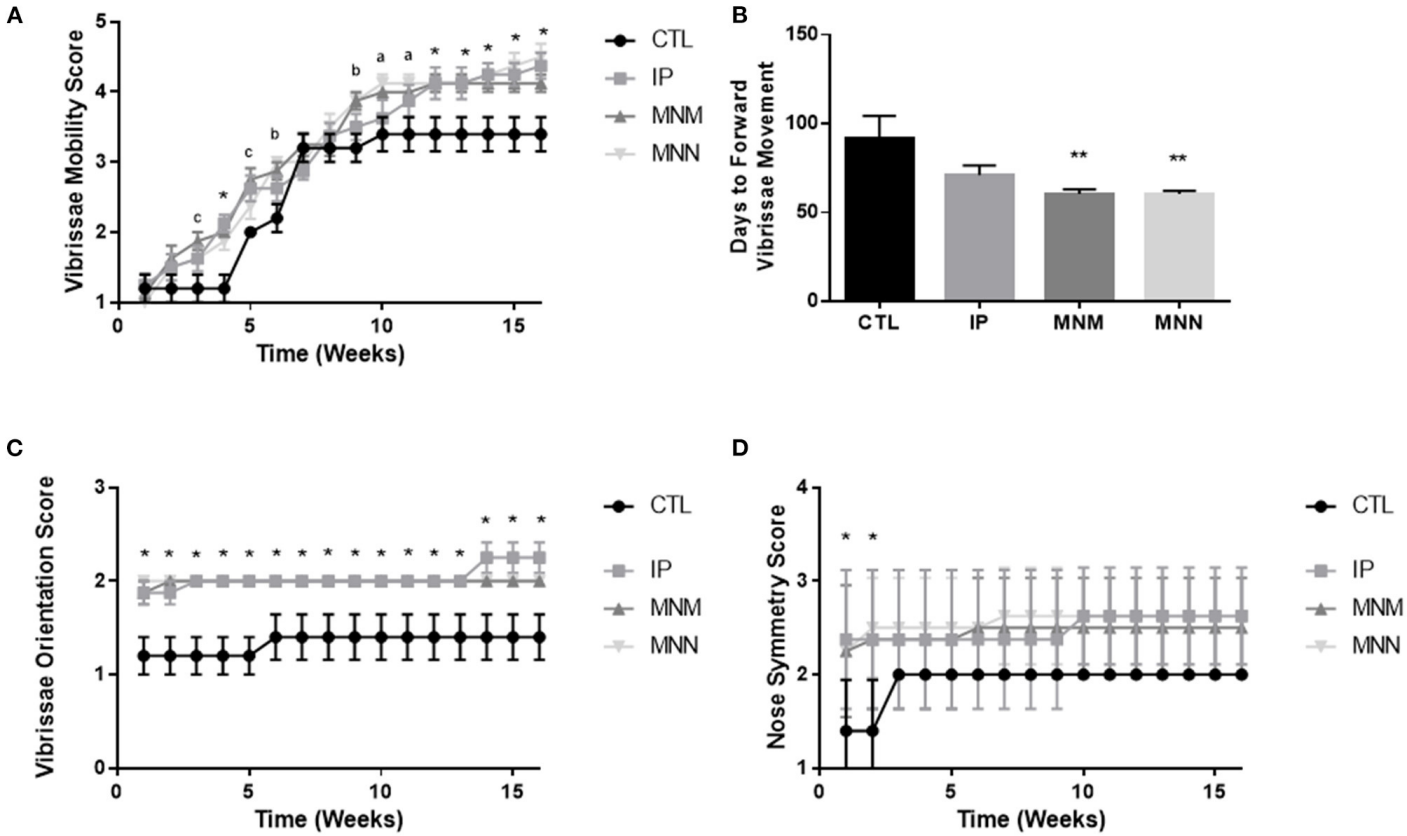
Functional recovery results: weekly vibrissae movement scores **(A)**, number of days until each group achieved forward vibrissae movement (defined as a score of 4 or above) **(B)**, weekly vibrissae orientation scores **(C)**, weekly snout symmetry scores **(D)**. Data represented as Mean ± SEM (**p < 0.01 as compared to CTL, *p < 0.05 all groups compared to control, ^a^*p* < 0.05 MNN alone as compared to CTL, ^b^*p* < 0.05 MNM and MNN as compared to CTL, and ^c^*p* < 0.05 MNN and IP as compared to CTL).

The effects of the grafting techniques on recovery of facial function was followed for 16 weeks following the surgical intervention. Figure 2A displays the significant improvement in vibrissae movement among all three intervention groups as compared to the CTL group. Significance in both grafting technique and time was shown by the two-way ANOVA. The multiple comparisons test revealed statistical significance amongst the three grafting techniques as compared to the CTL group (^**^p < 0.01 and ^*^p < 0.05 all groups compared to control, ^*a*^*p* < 0.05 MNN alone as compared to CTL, ^*b*^*p* < 0.05 MNM and MNN as compared to CTL, and ^*c*^*p* < 0.05 MNN and IP as compared to CTL). The MNN and MNM grafting groups had a significantly faster return of forward vibrissae movement (defined as a score of 4 or greater) when compared to the CTL group (^**^p < 0.01), with an average return of movement at 60.38 ± 1.84 and 60.38 ± 2.63 days as compared to 91.00 ± 13.28 days, respectively (Figure 2B). At the end of the 16 experiment, 50% of MNN animals, 38% of IP, and 13% of MNM, achieved coordinated vibrissae movement (defined as a score of 5 or greater) (Table 1). However, none of the CTL animals achieved coordinated vibrissae movement. All interventions significantly improved vibrissae orientation compared to the CTL (^*^p < 0.05) (Figure 2C). In terms of snout symmetry (assessed on a 4-point scale), all three intervention groups reached a mean score of ~2.5 while the control group reached a score of 2. Although not statistically significant at 16 weeks, all three intervention groups reached their final symmetry scores sooner than the control group (^*^p < 0.05) (Figure 2D).

**Table 1:** Percentage of animals per group achieving coordinated forward vibrissae movement (defined as a score of 5 or above) at 16 weeks.

**Control**	**Interposition graft**	**MNM graft**	**MNN graft**
0% (0/5)	37.5% (3/8)	12.5% (1/8)	50% (4/8)

### Muscle Atrophy

**Figure 3:**
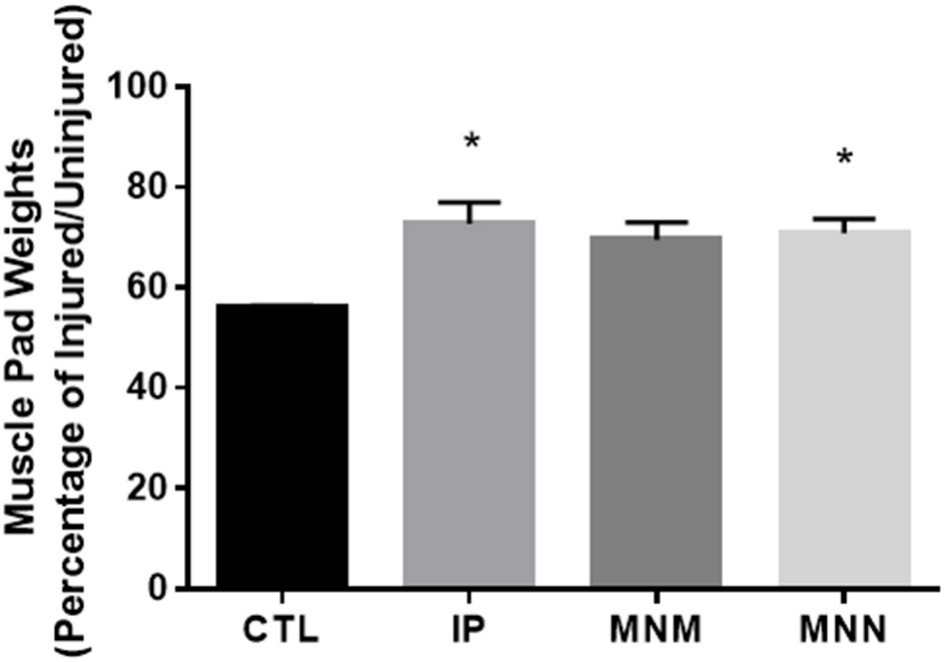
Mean vibrissae muscle pad weight in each group. Muscle atrophy was determined by calculating the denervated muscle pad weight as standardized to that of the innervated muscle pad. Data represented as Mean ± SEM (*p < 0.05).

To determine the effect of the grafting techniques on muscle atrophy, the muscle pads were dissected and weighed. The muscle pads of the CTL group, with no attempt at reinnervation, weighed 252.6 ± 28.5 milligrams (mg) or 55.86 ± 0.63% the size of the uninjured muscle pad weight at 16 weeks. A significant effect of the grafting techniques was determined by the ANOVA [*F*_(3, 28)_ = 1.990, *p* = 0.0076]. The multiple comparisons test revealed that he IP and MNN groups retained significantly more muscle pad weight at 346.8 ± 23.5 and 400.5 ± 14.4 mg or 72.69 ± 4.35 and 70.94 ± 2.78%, respectively (^*^p < 0.05 as compared to CTL) (Figure 3). Although the weight of the muscle pads from the MNM group increased to 428.7 ± 43.2 mg or 69.54 ± 3.49% of the uninjured muscle pad, this change was not significant.

## Discussion

Neurotization, the implantation of a nerve directly into a denervated muscle, was first described in the early 1900's by Hacker. Direct neurotization was initially explored for use of denervated muscles in poliomyelitis (12). This technique has proven successful in multiple animal studies, but its clinical use has only sporadically been described in the literature (12). Clinically, more conventional methods for reconstruction, including nerve grafting and nerve transfer techniques, have been employed. These conventional methods require either the presence of both ends of a severed nerve, or they utilize unrelated motor nerves that potentially provide muscle tone or learned muscle contractions.

In previous papers, we explored the method of direct neurotization using a muscle-nerve-muscle graft (5, 9). We demonstrated that an innervated muscle will sprout axons that enter and transverse the graft, and innervate a denervated muscle. In the current study, we describe the MNN grafting technique which is a variant to the MNM model in which one end of the nerve conduit is embedded into the normal muscle and the other end is anastomosed to the severed distal nerve that supplied the denervated muscle. Although IP grafting is typically the ideal surgical strategy, MNM and MNN grafting techniques have multiple advantages. IP grafting often times results in synkinesis and unpair movements. In paired, symmetrically functioning muscles such as in the face and larynx, the potential of symmetrical movement can be realized with the MNM and MNN grafting techniques. It can be highly advantageous to capitalize on the property of symmetric movements with unilateral deficits and use the uninjured side to supply the injured side with innervation or some form of signaling (13). Although independent, unilateral functioning would not occur with neural pairing of these symmetrical muscles, for most purposes, the motor deficit would be minimized.

Poor axonal regeneration across the injury site had been well-established in multiple peripheral nerve injury models and many studies have explored therapeutic agents, including the use of electrical stimulation, to improve the efficacy of axonal regeneration across the injury site (5, 14–18). The IP grafting technique requires two sutured sites to be traversed by the sprouting axons. Although the MNM and MNN grafting techniques does not eliminate the need for a harvest nerve graft, they do reduce the need for surgical neurorrhaphy. The MNM grafting technique eliminates all surgical neurorrhaphy while MNN grafting technique reduces the number of suture sites for the regenerating axon to transverse to only one. However, one limitation of the MNN and MNN techniques is that their success depends on sprouting axons from the donor muscle to enter the graft.

This paper is the first to demonstrate the use of muscle-nerve-nerve grafting as an alternative option to interpositional grafting. In this study, animals that had the MNN grafting repair recovered facial behavioral function similar to the animals that had the IP and MNM grafting repair techniques. The behavioral results from the IP and MNM grafting groups in this study were consistent with our previous findings (5, 9). These data suggest that in surgical situations when IP grafting may be challenging including trauma or tumor removal, the MNN grafting repair may be a promising alternative surgical option to gain symmetric axonal regeneration.

In our previous study, the lower zygomatic branch of the facial nerve was identified and noted to contribute to whisking outcomes (9). When the lower zygomatic branch was transected, animals did not achieve any recovery of facial function. In the current study, we did not transect this branch in an attempt to have earlier and greater recovery in the intervention groups. Allowing the lower zygomatic branch to remain intact lead to some recovery in the control group, most likely as a result of axonal compensation. However, the improvement in movement in the MNN or MNM groups compared to the control group in this paper is considered the contribution of the nerve graft(s). Since it was hypothesized that earlier and potentially greater recovery would be observed because the lower zygomatic branch remained intact in these animals, the 16- week experimental time course remained consistent with our prior studies (9). However, this study could have benefited from a longer experimental time course which to capture the overall potential of the grafting techniques.

Muscle atrophy was significantly decreased, and vibrissae movement was significantly improved in the IP and MNN grafting groups compared to the control group. However, most promising was the finding that the MNN grafting technique was comparable to the clinically widely accepted IP grafting technique in both attaining vibrissae movement as well as minimizing muscle atrophy. No difference was observed between the MNM and MNN grafting. We hypothesize that since the IP and MNN grafting techniques utilize the distal nerve as part of the nerve conduit, the original neuromuscular junctions have the potential to provide enhanced muscle reinnervation. Future studies will examine the histological differences in neuromuscular junction occupancy in all groups.

In some regard, the MNM and MNN techniques are new paradigms for reinnervation. These are the only models in which neural input for a denervated muscle is not coming directly from the nervous system. All other techniques utilize damaged, altered, or misfit inputs directly from the peripheral nervous system (PNS). Common otolaryngologic examples include the XII-VII grafting for facial nerve paralysis and the ansa hypoglossi-recurrent laryngeal nerve anastomosis for vocal cord paralysis (19–23). These nerve grafts normally innervate multiple, independently functioning muscles and thus bring misfit signals from the PNS to muscles whose function is completely different from the nerves' intended purpose. To the contrary, the MNM and MNN grafts bring signals directly from muscles, not from the PNS. Also, the signals they carry are simplified, only transmitting neural input from a single source that induces muscle contraction from a similar, single functioning muscle (the dilator naris muscle). Although this technique can lead to damage to the healthy, contralateral muscle pad, trauma to this area is minimal and no significant detrimental effects were noted in the contralateral whisking and snout function.

It may be reasoned that innervating a paralyzed muscle with the nerves from an intact muscle is essentially creating one functioning muscle from two. By doing so, contraction of the denervated muscle has the potential to be more specific, more natural, and stronger. Stimulation of the intact muscle would result in near simultaneous contraction of both muscles. Note that this idea is only an extension of what actually occurs when reinnervation of the distal denervated portion of a lacerated muscle transpires by ingrowth of nerves from the muscle's proximal intact nerve (18). Thus, the MNM and MNN techniques may also be considered when repairing lacerated muscles or when attaching muscle flaps to partially resected muscles. It is important to recognize that these techniques are unique in that they can be utilized in scenarios as described above in which a proximal nerve stump is not present. The MNM or MNN technique could create a muscle flap that potentially becomes a functional extension of the muscle from which it receives its graft.

Many questions remain regarding the efficacy and potential applications of MNM and MNN grafts. Future studies are needed to determine how long after muscle denervation the grafts will be effective and whether there is the limiting length of the graft. We will also explore possible neurotherapeutic strategies to enhance axonal sprouting from the innervated muscle as well as enhance axonal regeneration across the sutured site. Lastly, we will explore whether artificial grafts will function as well as autogenous ones.

## Conclusion

This is the first study to evaluate the efficacy of MNN neurotization for facial nerve injury. Our results suggest MNN grafting is a viable technique for repair of unilateral peripheral nerve paralysis. For patients with unilateral peripheral nerve injury, particularly those who are not candidates for primary neurorrhaphy, this study provides a promising adjuvant or alternative technique for reanimation and reinnervation. Future studies may explore MNN grafting in the larynx, smaller facial nerve branches, extremities, and other areas of denervation.

## Data Availability Statement

The raw data supporting the conclusions of this article will be made available by the authors, without undue reservation.

## Ethics Statement

The animal use protocol was approved by the Institutional Animal Care and Use Committee (IACUC) of Edward Hines Jr. VA Hospital.

## Author Contributions

SC, MH, and EF contributed to conception and design of the study. MH, SB, and JS performed the technical work and gather the data. EF performed the statistical analysis. SC wrote the first draft of the manuscript. MH, EF, and SC wrote sections of the manuscript. All authors contributed to manuscript revision, read, and approved the submitted version.

## Funding

Funding support from the Department of Otolaryngology—Head and Neck Surgery at Loyola Medical Center, Maywood, Illinois.

## Conflict of Interest

The authors declare that the research was conducted in the absence of any commercial or financial relationships that could be construed as a potential conflict of interest.

## Publisher's Note

All claims expressed in this article are solely those of the authors and do not necessarily represent those of their affiliated organizations, or those of the publisher, the editors and the reviewers. Any product that may be evaluated in this article, or claim that may be made by its manufacturer, is not guaranteed or endorsed by the publisher.
